# Long‐term response to afatinib in an elderly patient with uncommon epidermal growth factor receptor mutation‐positive lung adenocarcinoma

**DOI:** 10.1111/1759-7714.13869

**Published:** 2021-02-03

**Authors:** Naoki Shijubou, Toshiyuki Sumi, Koki Kamada, Takeyuki Sawai, Yuichi Yamada, Hisashi Nakata, Yuji Mori, Hirofumi Chiba

**Affiliations:** ^1^ Department of Respiratory Medicine Hakodate Goryoukaku Hospital Hakodate‐shi Japan; ^2^ Department of Respiratory Medicine and Allergology Sapporo Medical University School of Medicine Sapporo‐shi Japan

**Keywords:** afatinib, elderly, lung cancer

## Abstract

Epidermal growth factor receptor (EGFR) tyrosine kinase inhibitors are the standard treatment for patients with non‐small cell lung cancer (NSCLC) harboring *EGFR* mutations. Uncommon mutations, excluding exon 19 deletions and exon 21 L858R, comprise 7%–23% of *EGFR* mutation‐positive NSCLC. The treatment of uncommon *EGFR* mutation‐positive NSCLCs is controversial. Here, we present the case of an 81‐year‐old man who was diagnosed with lung adenocarcinoma cStage IVA harboring the uncommon *EGFR* L861Q mutation. The patient received oral afatinib treatment (40 mg/day). One month after the initiation of afatinib treatment, Common Terminology Criteria for Adverse Events version 4.0 grade 2 stomatitis was observed. It improved upon afatinib withdrawal. After 10 days of withdrawal, afatinib treatment was resumed at a reduced dose of 20 mg/day. Subsequently, the patient continued treatment with afatinib. A partial response to afatinib treatment was maintained for 49 months until primary tumor regrowth. Afatinib treatment was continued after disease progression, but the patient died of bacterial pneumonia 59 months after initiation of afatinib treatment. Several studies have previously reported a large number of compound mutations with uncommon mutations, and that compound mutation‐induced cells are most susceptible to afatinib. This suggests the efficacy of afatinib in clinical practice and that afatinib may be safely administered to elderly patients with appropriate dose reductions.

## Graphical abstract

A large number of compound mutations have been observed in patients with uncommon *EGFR* mutation‐positive NSCLC. Compound mutation‐induced cells are most susceptible to afatinib, suggesting the efficacy of afatinib in clinical practice. Afatinib may be administered to elderly patients with appropriate dose adjustments

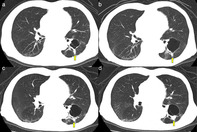

## INTRODUCTION

Epidermal growth factor receptor (EGFR) tyrosine kinase inhibitors (TKIs) are the standard treatment for patients with non‐small cell lung cancer (NSCLC) harboring *EGFR* mutations.^1^, ^2^, ^3^ Common mutations account for the majority of *EGFR* mutation‐positive NSCLC. However, 7%–23% of *EGFR* mutation‐positive NSCLC, except for exon 19 deletions and exon 21 L858R, are uncommon mutations.^4^ Retrospective studies discovered that uncommon *EGFR* mutation‐positive NSCLC developed early resistance to first‐generation EGFR‐TKIs. Afatinib, a second‐generation EGFR‐TKI, was found to have a clinical benefit for patients with NSCLC harboring uncommon *EGFR* mutations.^5^ Osimertinib, a third‐generation TKI, was also found to be clinically effective for treating uncommon *EGFR* mutation‐positive NSCLC.^6^ Treatment of uncommon *EGFR* mutation‐positive NSCLC is controversial. Moreover, the proportion of elderly participants in clinical trials is small, therefore the safety of EGFR‐TKIs, such as afatinib, in these patients remains unclear.^7^

Here, we report a case of a long‐term response to afatinib in an elderly patient harboring uncommon *EGFR* mutation‐positive lung adenocarcinoma.

## CASE REPORT

**FIGURE 1:**
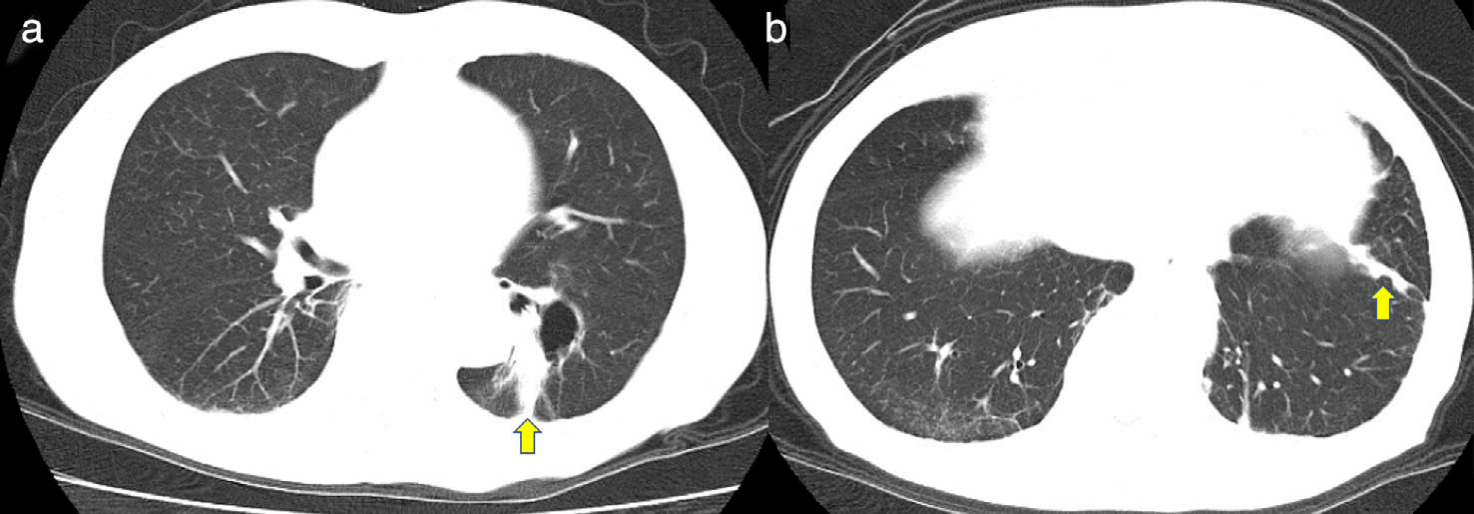
Chest computed tomography (CT) findings before the start of afatinib treatment. The yellow arrows indicate the tumor adjacent to the cyst (a) and pleural dissemination (b)

An 81‐year‐old man was referred to our hospital following chest xray which revealed an abnormal shadow, and he was subsequently diagnosed with lung adenocarcinoma cT1bN0M1a (M: PLE) Stage IVA (Figure 1).

**FIGURE 2:**
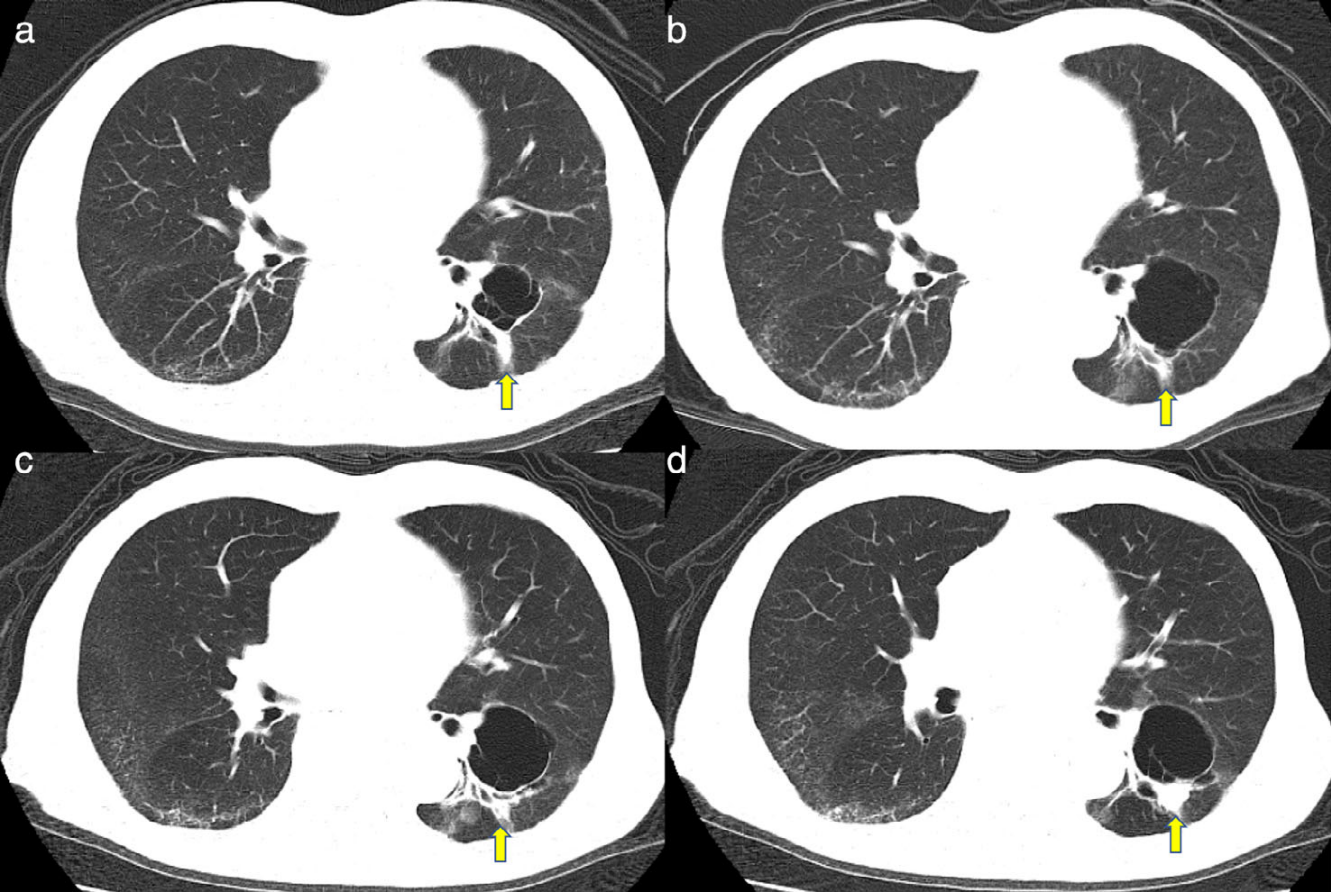
The clinical course of chest computed tomography (CT) findings. (a) The tumor shrunk three months after afatinib administration. (b) The antitumor effect of afatinib was maintained in partial response at 18 months and (c) 42 months after the start of afatinib treatment. (d) The tumor was enlarged 49 months after the initiation of afatinib. The yellow arrows indicate the primary tumor

Examination of the DNA sequence of the *EGFR* gene revealed an uncommon *EGFR* L861Q mutation. The patient received treatment with afatinib administered orally (40 mg/day). One month later, Common Terminology Criteria for Adverse Events (CTCAE) version 4.0 grade 2 stomatitis was observed, which improved with afatinib withdrawal. After 10 days of withdrawal, afatanib treatment was resumed at a reduced dose of 20 mg/day. Subsequently, grade 1 skin toxicity was observed. However, the patient continued treatment with afatinib. A partial response to afatinib treatment was noted for 49 months until the primary tumor recurred (Figure 2). Treatment with afatinib was continued after disease progression, and he died of bacterial pneumonia 59 months after the initiation of afatinib treatment. The patient provided oral informed consent for the publication of this report.

## DISCUSSION

The elderly patient in our report, who presented with lung adenocarcinoma with an uncommon *EGFR* mutation, developed a long‐term response to afatinib with appropriate dose reduction.

An integrated analysis of LUX‐Lung 2, 3, and 6 reported the clinical benefits of afatinib, a second‐generation TKI, for treating *EGFR* mutation‐positive NSCLC with uncommon mutations.^5^ The objective response rate (ORR) was 71.1%, and the median duration of response (mDoR) was 11.1 months among patients with lung adenocarcinoma harboring uncommon *EGFR* mutations, namely G719X, L861Q, and S761I.^5^ The ORR for *EGFR* mutation‐positive NSCLC with L861Q was 56.3%. Afatinib has previously been associated with an ORR of 59.6% in *EGFR* mutation‐positive NSCLC with L861Q.^4^ It has been reported that 6.8% of patients with major uncommon *EGFR* mutation‐positive NSCLC responded to afatinib for more than three years. In our case, the patient remained responsive for more than four years.^4^ On the other hand, osimertinib has also been reported to have an ORR of 50% and mDoR of 9.8 months for treating *EGFR* mutation‐positive NSCLC patients with major uncommon *EGFR* mutations.^6^ The frequency and pattern of compound mutations in *EGFR* mutations including L858R/del19, G719C/S/A, and L861Q mutations have been reported in 15.9%, 93.3%, and 36.4% of all cases, respectively.^8^ Upon analyzing the association between *EGFR* mutation and resistance, there was no difference between afatinib and osimertinib in terms of the susceptibility of uncommon mutation‐induced cells to them. However, compound mutation‐induced cells have been reported to be most susceptible to afatinib.^8^ A large number of compound mutations have been observed in uncommon mutations, suggesting the efficacy of afatinib. Cells carrying the EGFR L861Q mutation have been reported to be less sensitive to EGFR‐specific inhibitors, but more sensitive to pan ERBB inhibitors. This suggests that afatinib may be effective in treating NSCLC harboring EGFR L861Q mutations.^9^

Since the proportion of elderly participants in clinical trials is small, the safety of the administration of EGFR‐TKIs, including afatinib, in the elderly population remains unclear. In the LUX‐Lung 3 and 6 analysis, the rate at which patients required dose reduction was higher in the afatinib group. However, the trend was similar among younger and elderly patients.^10^ Treatment‐related adverse events are often associated with afatinib dose reductions, regardless of age. Low discontinuation rates with appropriate dose reduction protocols, and dose reductions which have reduced the incidence of grade > 3 AEs but have not significantly altered the treatment effect have been previously reported.^11^ There have also been reports of clinical trials starting afatinib at low doses in patients harboring common *EGFR* mutations, which have shown promising clinical efficacy and good tolerability. A phase II study using low starting doses of afatinib reported that 22% of patients aged 75 years or older who started with afatinib at 20 mg/day were able to increase the dose up to 30 mg/day, and 17% were able to increase the dose to 40 mg/day, with the majority at 20 mg/day.^12^ Although the number of patients was small, a phase I study investigating the optimal dose of afatinib in elderly patients recommended 30 mg/day,^13^ and other phase II studies also showed that afatinib 30 mg/day was effective and feasible in elderly patients.^14^ For the elderly, 20–30 mg/day is considered to be an appropriate dose. Although most reports of low‐dose afatinib are for common mutations, there has been a study reporting that low‐dose afatinib can be safely used without reducing its efficacy in elderly patients harboring uncommon mutations.^15^ In this case, an appropriate reduction in the dose of afatinib resulted in a long‐term response. This suggests that the response to treatment could be maintained in elderly patients without the need to discontinue treatment due to adverse events.

In conclusion, afatinib is effective in treating NSCLC harboring uncommon *EGFR* mutations, and may be administered safely to elderly patients with an appropriate dose reduction.

## CONFLICT OF INTEREST

All authors declare that they have no conflicts of interest.
